# From Static Outputs to Living Evidence: AI for Integrated Knowledge Translation in Canadian Health Research

**DOI:** 10.2196/99386

**Published:** 2026-07-31

**Authors:** Zack van Allen, Jayne Beselt, Jerry M Maniate, Samuel Hickcox, Joshua A Rash, Kumanan Wilson, Douglas Archibald, Arun Radhakrishnan

**Affiliations:** 1 Bruyere Health Research Institute, 85 Primrose Ave, Ottawa, ON, K1R 6M1, Canada, 1 (613) 562-6262; 2 Department of Family Medicine, University of Ottawa, Ottawa, ON, Canada; 3 Department of Innovation in Medical Education, University of Ottawa, Ottawa, ON, Canada; 4 Department of Medicine, Faculty of Medicine, University of Ottawa, Ottawa, ON, Canada; 5 Office of Addictions and Mental Health, Nova Scotia Department of Health and Wellness, Halifax, NS, Canada; 6 Department of Psychology, Memorial University of Newfoundland, St. John's, NL, Canada; 7 Department of Psychology and Health Studies, University of Saskatchewan, Saskatoon, SK, Canada

**Keywords:** integrated knowledge translation, knowledge mobilization, large language models, retrieval-augmented generation, evidence synthesis, health policy, data governance

## Abstract

Integrated knowledge translation still relies on static reports, presentations, and manuscripts that cannot adapt to decision-makers’ evolving questions. Retrieval-augmented large language models can add a secure, auditable conversational layer over curated program outputs and selected research materials, enabling rapid, traceable synthesis between meetings and across portfolios. Treating these tools as governed infrastructure, with mandatory provenance, privacy protections, transparent documentation, and equity by design, could help reduce friction in evidence exchange and shorten the lag between knowledge creation and use. This viewpoint paper advances a conceptual design vision and the governance it requires, rather than reporting an evaluation of a deployed system.

## Current State of Knowledge Translation

AI is reshaping many parts of medicine and health research. Most discussions have focused on clinical applications, but an underexplored question is how AI can support knowledge translation (KT). In this viewpoint paper, we argue that retrieval-augmented large language models (LLMs) can help integrated KT (iKT) move beyond static end-of-grant products and toward more interactive, governed, and auditable evidence use. Our aim is conceptual and practice oriented: we describe a design vision and its governance requirements, not an evaluation of an implemented system.

KT is an iterative process that includes synthesis, dissemination, exchange, and the ethically sound application of knowledge [1]. iKT is a partnership approach in which knowledge users (such as clinicians, policymakers, health care administrators, community leaders, and patients) are engaged throughout the research process, not only at the end of a study [1,2]. In the Canadian context, *knowledge mobilization* is often used as a broader umbrella term for these synthesis, exchange, and application activities, whereas *integrated knowledge translation* specifies the partnered coproduction approach we focus on here. In practice however, many KT interactions still depend on static artifacts produced at the end of a project, including manuscripts, slide decks, and final reports. This creates a paradox: coproduced research and synthesis products answer specific questions at a given point in time, yet they do not adapt well as policy questions evolve. Further interrogation of the underlying evidence is often impractical. Knowledge users rarely have time to synthesize insights across dozens of documents, and research teams rarely have the capacity to manually tailor responses to every emerging question.

## A Conversational Layer Over Evidence: “Chat With the Data”

LLMs make it feasible to build KT workflows that support conversational access to evidence. An LLM-enabled KT process would allow knowledge users and research teams to interrogate curated corpora by using natural language. The aim is to reduce the friction of finding, integrating, and explaining evidence while preserving an auditable trail to source material, without outsourcing judgment to a model. Herein, “chatting with the data” refers to interaction with a governed interface over curated, permissioned, and documented sources, where the inclusion of the underlying raw data is optional and depends on the nature of the project.

**Figure 1.**
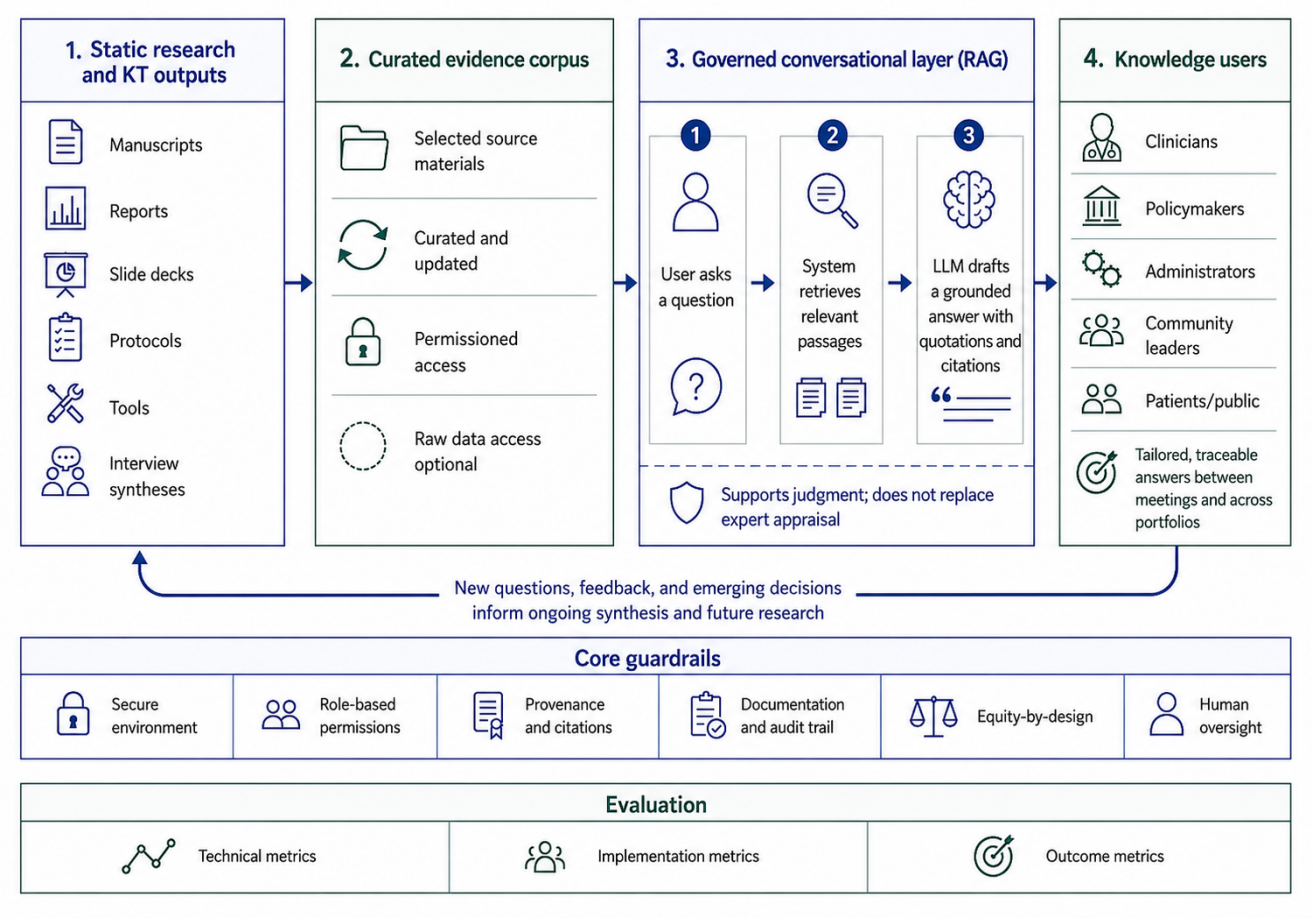
Conceptual workflow for conversational knowledge translation (KT), from static research and KT outputs through a curated evidence corpus and a governed conversational layer (retrieval-augmented generation [RAG]) to knowledge users, with core guardrails and evaluation domains. LLM: large language model.

In practice, this is increasingly implemented through retrieval-augmented generation (RAG), in which a system retrieves relevant passages from a defined corpus and constrains model output to those materials [3]. A user asks a question; the system searches the underlying corpus (eg, publications, protocols, tools, progress reports, or interview transcripts), and the LLM drafts an answer grounded in the retrieved passages. The corpus need not be limited to primary materials; it can also include the analyzed and synthesized outputs derived from them, thereby allowing answers to point to externally visible, shareable products, such as published syntheses, reports, or dashboards, even when some of the underlying data remain access restricted. When designed well, the interface shows quotations and citations by default and allows users to open the surrounding context with minimal effort. Tailored answers paired with grounded citations can shift KT from one-way dissemination toward an interactive dialogue anchored in the underlying evidence. Crucially, this conversational approach can also help unlock the value of health research data that are typically inaccessible once the final synthesis is completed, thereby enabling ongoing engagement with evidence that might otherwise remain siloed in archived datasets and reports. RAG improves traceability and grounding, but it does not eliminate hallucination, retrieval failure, or interpretive bias, and citation-backed answers can convey more authority than what the underlying evidence warrants. Such systems should therefore support—not replace—expert appraisal by using interface cues that prompt users to verify claims against the cited source text rather than accept them at face value. Figure 1 summarizes this conceptual workflow, including the core guardrails and evaluation domains.

Ongoing two-way interactions between knowledge users and researchers are complicated by familiar iKT barriers, including limited time, limited resources, and turnover among decision-makers that can disrupt continuity. A conversational agent can serve as a digital complement to knowledge brokers, supporting evidence exchange between meetings and across research portfolios [4]. It can also generate useful feedback signals; the questions that users ask and the places where the corpus cannot answer them can reveal information needs, implementation constraints, and evidence gaps that should shape the next research cycle.

## Supporting iKT Across Research Programs

The opportunity for AI-enabled iKT is not limited to single studies. Funders and research coordinating hubs manage portfolios of projects with shared goals and overlapping knowledge users. For example, the Canadian Institutes of Health Research (CIHR) Strengthening the Health Workforce for System Transformation initiative includes a Health Workforce Research Evidence Support and Knowledge Mobilization Hub with an explicit mandate to help funded teams bridge the evidence-policy-practice gap, promote knowledge mobilization, foster networking, and coordinate rapid responses between research teams and knowledge users [5].

Yet, the current flow of portfolio-level knowledge often resembles a flood of static documents—manuscripts, annual progress reports, meeting minutes, slide decks, rapid syntheses, and final reports. These outputs are valuable, but they are difficult to navigate, search, and integrate across projects. They also are not usually packaged in ways that directly match the evolving questions of decision-makers.

A conversational system can index and link these artifacts, allowing users to ask questions, such as “what have we learned so far about rural recruitment” or “which projects have evidence on retention interventions,” and receive answers with traceable links to the underlying sources. This supports rapid synthesis and decision support without requiring every user to read every report.

## Study-Level iKT: Enabling Cointerpretation of Qualitative Evidence

At the project level, conversational tools can deepen engagement with qualitative evidence. An example is the Adaptive Mentoring Networks project within the previously mentioned CIHR initiative, which aims to study mentoring networks across Canada to address shortages, attrition, and burnout [6]. iKT partners, such as clinical leaders, educators, and professional associations, often have practical questions long before a conventional thematic paper is available. A secure, deidentified chat interface could let partners explore the data responsibly by asking “what do participants describe as the active ingredients of successful mentoring networks” or “how do experiences differ between primary care and other settings?”

Instead of waiting for a final manuscript, stakeholders could cointerpret emerging themes as analysis proceeds. They could flag overlooked areas, propose alternative interpretations, and help prioritize what should be drafted next. This would make iKT more iterative and responsive, as well as more useful to knowledge users, who often need evidence faster than what traditional publication cycles allow.

## Living Synthesis Across Mixed Qualitative Sources

Many KT questions require integration across multiple qualitative studies, local evaluations, and peer-reviewed evidence. Conversational systems can index mixed sources (published work plus selected underlying transcripts or field notes) and return synthesized answers while still showing the origins of the response. When done carefully, this starts to resemble a living synthesis that can be updated as new studies and outputs emerge, potentially reducing the lag between knowledge creation and use [7]. We use the term *living evidence* broadly, referring to continuously updated, query-responsive evidence infrastructure rather than the formal sense of living systematic review methodology. If designed well, these systems can also make blind spots visible; repeated, unanswered questions about equity impacts or rural contexts should trigger the collection of new data, not overconfident generalization.

## Guardrails: Privacy, Provenance, and Accountability

Four design principles should guide conversational KT. First, it should be treated as core infrastructure, not as an informal use of public chat tools. These systems may involve sensitive or unpublished information; therefore, they should operate in secure, closed environments with safeguards that prevent the inappropriate reuse of data, and they should define access levels for different users. Treating these systems as infrastructure implies nontrivial investment, including secure technical environments, ongoing curation and updating of the evidence corpus, and designated responsibility for monitoring and oversight, which in turn requires upskilling among KT practitioners and research teams. In part, this is a shift in where effort is directed rather than the addition of a wholly new burden; instead of repeatedly producing static end-of-grant materials, teams would invest in curating, updating, and monitoring a shared evidence resource. Although this is a substantial investment, we believe it is warranted. By lowering the effort required to find, integrate, and apply evidence, a governed conversational layer can make existing research and synthesis products meaningfully more usable—in general and for a larger number of knowledge users—than static outputs alone.

Second, provenance should be mandatory. The system should ground answers in retrieved text, display citations, and abstain when support is weak.

Third, developers and research teams should provide clear documentation about how the system works and where it may fail. Similar to reflexivity in qualitative research, this documentation should explain key design choices; what information is included or excluded; and common failure modes, such as confident-sounding answers that miss crucial context.

Fourth, equity should be designed into the system from the outset. This includes bilingual support where appropriate and governance that respects community data stewardship, including ownership, control, access, and possession (OCAP) principles when First Nations data are involved [8]. Equity by design also requires attention to whose evidence is represented in the corpus; who is authorized to ask questions; and how knowledge users, particularly those from marginalized groups, can contest or correct outputs. Differences in infrastructure access and digital literacy across settings should be anticipated, so that conversational KT narrows rather than widens existing asymmetries in whose questions shape evidence use. Conversely, a conversational layer that can synthesize and present evidence in the form that best fits each user (eg, by language, format, or level of detail) is itself potentially equity-advancing, broadening meaningful access for users who are poorly served by dense, static outputs. Some of these access disparities, with regard to both AI-enabled KT tools and the resources needed to add a conversational layer to one’s own data, could be mitigated through institutional and public-sector investment that supports these services on both the researcher side and the knowledge-user side.

Table 1 maps these principles to specific governance domains, the risks they address, example safeguards, and metrics by which each could be evaluated.

**Table 1.** Governance domains for conversational knowledge translation systems, with associated risks, example safeguards, and example evaluation metrics.

Governance domain	Risk addressed	Example safeguard	Example evaluation metric
Data access and role-based permissions	Inappropriate exposure of sensitive or unpublished data	Tiered access by source type, with stricter controls on unpublished transcripts than on published reports; closed environment	Appropriateness of access controls; number of unauthorized access events
Consent and secondary use	Reuse of participant data beyond what was agreed	Consent language and data management plans that anticipate governed AI reuse; participants can opt out	Proportion of corpus with AI reuse–compatible consent
Provenance and citation verification	Unsupported or overauthoritative answers	Mandatory grounding in retrieved text; displayed citations; abstention when support is weak	Citation accuracy; retrieval recall; appropriateness of abstention
Logging and audit trails	Without logs of queries, retrieved sources, and generated answers, erroneous or hallucinated outputs can go undetected and cannot be traced or audited, weakening accountability	Query and retrieval logging; human review of flagged or high-stakes outputs	Auditability; error detection rate
Data retention and residency	Vendor access and jurisdictional breach	On-premise or closed hosting; defined retention limits; no third-party model training on the corpus	Compliance with data residency and retention requirements
Accountability for errors	Diffuse responsibility when outputs mislead	Named data steward or owner; documented correction workflow	Time to correction; resolution of contested outputs
Equity by design	Reinforcing access and power asymmetries	Bilingual support; OCAP^a^ principles for First Nations data; mechanisms for users to contest outputs	Usability across knowledge-user groups; whose questions are answered

a OCAP: ownership, control, access, and possession.

## A Pragmatic Call to Action

AI will not fix KT by itself, but “chatting with the data” can reduce friction in finding, translating, and sustaining evidence exchange across networks. For health researchers, a practical next step is to design data collection processes and documentation for conversational reuse at the planning stage. This means budgeting for secure infrastructure and ensuring that research ethics board applications, consent forms, and data management plans allow data processing within governed AI environments. Research ethics approval is likely to be the most significant practical barrier. Teams should anticipate limits on the secondary use of data within AI systems; the difficulty of maintaining deidentification in interactive querying; and the need for consent language and data management plans that explicitly contemplate governed, AI-enabled reuse, including the option for participants to decline such reuse and clear terms that cover data residency and vendor access. For funders and coordinating hubs, the opportunity is to pilot secure conversational portfolios that allow programs to learn across projects while keeping evidence traceable. Evaluation should span the following three levels: technical metrics, such as citation accuracy, retrieval recall, and the appropriateness of abstention; implementation metrics, such as acceptability, feasibility, usability across different knowledge-user groups, and time saved; and outcome metrics, such as influence on decision-making, evidence uptake in policy or practice, and whether recurring unanswered questions inform future research priorities. The primary responsibility for this evaluation would rest with the institutions that host and operate these platforms, and they would work in partnership with the research teams and knowledge users they serve.

## Implications for the Health Information Ecosystem

The case for AI-enabled KT is broader than any single research program. Emerging survey evidence suggests that public interest in using generative AI for health information is already substantial, even as adoption, which is shaped by perceived usefulness, convenience, and digital access, remains uneven [9]. This creates both risks and opportunities. LLMs can amplify misinformation or hallucinated claims in high-stakes settings [10], yet conversational AI may also influence trust and attitudes toward scientific information under some conditions [11]. If public use of these tools continues to expand, health research organizations and funders should think not only about publication outputs but also about how trustworthy, traceable research findings will surface within AI-mediated information environments. This is precisely the capability that governed, provenance-first iKT infrastructure is intended to provide. When research findings carry their citations and context with them, they are more likely to surface reliably within these AI-mediated environments. Reimagining KT for this setting is increasingly urgent.

## Abbreviations

CIHR: Canadian Institutes of Health Research
iKT: integrated knowledge translation
KT: knowledge translation
LLM: large language model
OCAP: ownership, control, access, and possession
RAG: retrieval-augmented generation
